# 1,3-Bis[(4-nitro­benzyl­idene)amino­oxy]propane

**DOI:** 10.1107/S1600536809031316

**Published:** 2009-08-12

**Authors:** Wen-Kui Dong, Yin-Xia Sun, Jun-Feng Tong, Hai-Hong Zhao, Li Wang

**Affiliations:** a School of Chemical and Biological Engineering, Lanzhou Jiaotong University, Lanzhou 730070, People’s Republic of China

## Abstract

The complete molecule of title compound, C_17_H_16_N_4_O_6_, is generated by a crystallographic twofold axis. Within the mol­ecule, the two benzene units are approximately perpen­dicular, making a dihedral angle of 85.91 (4)°. In the crystal, mol­ecules are linked into a three-dimensional network by C—H⋯O hydrogen bonds and short O⋯O and N⋯O inter­actions, with distances of 2.998 (2) and 2.968 (3) Å, respectively.

## Related literature

For general background to Schiff base complexes and their applications, see: Niederhoffer *et al.* (1984 ▶); Zhang *et al.* (1990 ▶); Tisato *et al.* (1994 ▶); Lacroix (2001 ▶); Sundari *et al.* (1997 ▶); Koehler *et al.* (1964 ▶); Cordes & Jencks (1962 ▶); Akine *et al.* (2006 ▶). For related structures, see: Fun *et al.* (2008*a* ▶,*b* ▶); Kia *et al.* (2009 ▶); Shi *et al.* (2007 ▶); Ren *et al.* (2008 ▶); Ding *et al.* (2009 ▶); Dong *et al.* (2008*a* ▶). For a related Schiff base bis­oxime compound synthesized using a similar route, see: Dong *et al.* (2008*b* ▶).
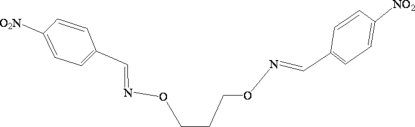


## Experimental

### 

#### Crystal data

C_17_H_16_N_4_O_6_
*M* *_r_* = 372.34
Monoclinic, 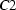

*a* = 29.005 (3) Å
*b* = 4.7878 (5) Å
*c* = 6.3579 (7) Å
β = 99.144 (1)°
*V* = 871.71 (16) Å^3^
*Z* = 2
Mo *K*α radiation
μ = 0.11 mm^−1^
*T* = 298 K
0.45 × 0.17 × 0.06 mm

#### Data collection

Siemens SMART 1000 CCD area-detector diffractometer
Absorption correction: multi-scan (*SADABS*; Sheldrick, 1996 ▶) *T*_min_ = 0.952, *T*_max_ = 0.993
2321 measured reflections
872 independent reflections
675 reflections with *I* > 2σ(*I*)
*R*_int_ = 0.049

#### Refinement

*R*[*F* ^2^ > 2σ(*F* ^2^)] = 0.039
*wR*(*F* ^2^) = 0.094
*S* = 0.96
872 reflections
123 parameters
1 restraint
H-atom parameters constrained
Δρ_max_ = 0.13 e Å^−3^
Δρ_min_ = −0.19 e Å^−3^

### 

Data collection: *SMART* (Siemens, 1996 ▶); cell refinement: *SAINT* (Siemens, 1996 ▶); data reduction: *SAINT*; program(s) used to solve structure: *SHELXS97* (Sheldrick, 2008 ▶); program(s) used to refine structure: *SHELXL97* (Sheldrick, 2008 ▶); molecular graphics: *SHELXTL* (Sheldrick, 2008 ▶); software used to prepare material for publication: *SHELXTL*.

## Supplementary Material

Crystal structure: contains datablocks global, I. DOI: 10.1107/S1600536809031316/fl2256sup1.cif

Structure factors: contains datablocks I. DOI: 10.1107/S1600536809031316/fl2256Isup2.hkl

Additional supplementary materials:  crystallographic information; 3D view; checkCIF report

## Figures and Tables

**Table 1:** Hydrogen-bond geometry (Å, °)

*D*—H⋯*A*	*D*—H	H⋯*A*	*D*⋯*A*	*D*—H⋯*A*
C3—H3⋯O3^i^	0.93	2.40	3.206 (4)	145
C9—H9⋯O3^i^	0.93	2.63	3.395 (4)	139
C9—H9⋯O2^ii^	0.93	2.71	3.374 (4)	129

Symmetry codes: (i) 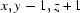; (ii) 
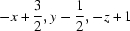
.

**Fig. 1.**
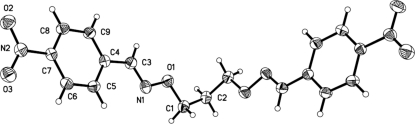
The molecular structure of the title compound with atom numbering scheme. Displacement ellipsoids for non-hydrogen atoms are drawn at the 30% probability level.

**Fig. 2.**
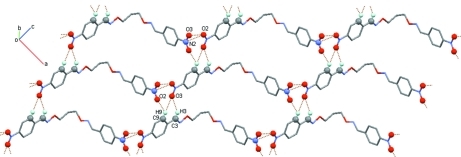
The intermolecular hydrogen bonds and short O···O and N···O interactions (dashed lines), showing zigzag chains along a axis and wave-like layers parallel to the ac plane.

**Fig. 3.**
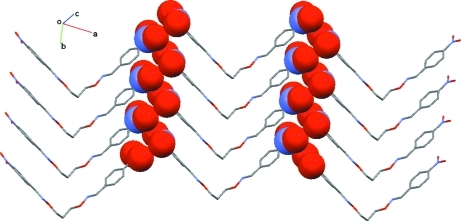
The infinite helical chains along b axis linked by short O···O and N···O interactions, other atoms are omitted for clarity.

**Fig. 4.**
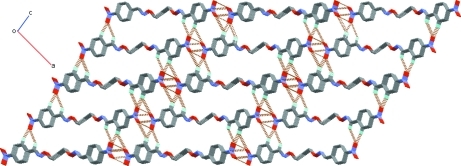
Part of the three-dimensional supramolecular network structure of the title compound.

## supplementary crystallographic information

### Comment

Schiff bases are among the most prevalent mixed-donor ligands in the field of coordination chemistry in which there has been growing interest, mainly because of their wide application in areas such as biochemistry (Niederhoffer *et al.*, 1984), catalysis (Zhang *et al.*, 1990), medical imaging (Tisato *et al.*, 1994), optical materials (Lacroix, 2001) and thin films (Sundari *et al.*, 1997). Although most Schiff bases are stable in both solution and the solid state, C=N bonds often suffer exchange reactions (Koehler *et al.*, 1964) as well as hydrolysis (Cordes & Jencks, 1962). Rate constants of oxime formation are smaller than those of imine formation and the equilibrium constants are larger by several orders (Akine *et al.*, 2006). Hence, the title compound should be stable enough to resist the metathesis of the C=N bonds. Many bisdentate Schiff base compounds have been structurally characterized (Fun *et al.*, 2008*a*; Fun *et al.*, 2008*b*; Kia *et al.*, 2009), but only a relatively small number of bisoxime compounds have had their X-ray structures reported (Shi *et al.*, 2007; Ren *et al.*, 2008). As an extension of our work (Ding *et al.*, 2009; Dong *et al.*, 2008*a*) on the structural characterization of bisoxime compounds, the title compound, is reported here (Fig. 1).

In the title compound all bond lengths are in normal ranges. The molecule sits on a crystallographic twofold passing through the central CH_2_ group (symmetry code: -*x*, *y*, -*z*) such that there is 1/2 molecule per asymmetric unit. Within the molecule, the dihedral angle between the plane of oxime functional group and benzene ring is about 0.54 (3)° for O1—N1—C3 and the C4—C9 ring, and the two benzene rings are approximately perpendicular with a dihedral angle of 85.91 (4)°. In the crystal intermolecular C—H···O hydrogen bonds link the molecules into an infinite three-dimensional supramolecular network. The molecules are held together by intermolecular hydrogen bonds (Table 1) to form infinite zigzag chains along the *a* axis and wave-like layers parallel to the *ac* plane (Fig. 2). In addition, the interesting features of the crystal structure are short intermolecular O···O and N···O interactions that form infinite helical chains along the *b* axis as depicted in Fig. 3. The O···O and N···O distances of 2.998 (2) and 2.968 (3) Å, respectively, are significantly shorter than the sum of the van der Waals radii of the relevant atoms. Thus, the zigzag and helical chains form a three-dimensional supramolecular structure through the crosslinked hydrogen-bonded and short intermolecular O···O and N···O interactions (Fig. 4).

### Experimental

The title compound was synthesized according to an analogous method reported earlier (Dong *et al.*, 2008*b*). To an ethanol solution (2 ml) of *p*-nitrobenzene (186.6 mg, 1.235 mmol) was added dropwise an ethanol solution (3 ml) of 1,3-bis(aminooxy)propane (51.3 mg, 0.473 mmol). The mixture was stirred at 328 K for 3 h. After cooling to room temperature, the precipitate was filtered off, and washed successively with ethanol and n-hexane, respectively. The product was dried *in vacuo* and purified by recrystallization from ethanol to yield 127.8 mg of (1); Yield, 71.8%. m. p. 427–429 K. Anal. Calcd. for C_17_H_16_N_4_O_6_: C, 54.84; H, 4.33; N, 15.05; Found: C, 55.06; H, 4.29; N, 15.04.

Colorless needle-like single crystals suitable for X-ray diffraction studies were obtained after about two weeks by slow evaporation from a chloroform-*N*,*N*-dimethylformamide of mixed solution of the title compound at room temperature.

### Refinement

Non-H atoms were refined anisotropically. H atoms were treated as riding atoms with distances C—H = 0.97 (CH_2_) and 0.93 Å (CH), and *U*_iso_(H) = 1.2 *U*_eq_(C) and 1.5 *U*_eq_(O).Since it was not possible to determine the absolute configuration of the molecule from the experimental data the Friedel equivalents were merged prior to final refinement cycles.

### Crystal data

**Table:**

C_17_H_16_N_4_O_6_	*F*(000) = 388
*M**_r_* = 372.34	*D*_x_ = 1.419 Mg m^−^^3^
Monoclinic, *C*2	Melting point = 427–429 K
Hall symbol: C 2y	Mo *K*α radiation, λ = 0.71073 Å
*a* = 29.005 (3) Å	Cell parameters from 773 reflections
*b* = 4.7878 (5) Å	θ = 2.9–25.3°
*c* = 6.3579 (7) Å	µ = 0.11 mm^−^^1^
β = 99.144 (1)°	*T* = 298 K
*V* = 871.71 (16) Å^3^	Needle-like, colorless
*Z* = 2	0.45 × 0.17 × 0.06 mm

### Data collection

**Table:**

Siemens SMART 1000 CCD area-detector diffractometer	872 independent reflections
Radiation source: fine-focus sealed tube	675 reflections with *I* > 2σ(*I*)
graphite	*R*_int_ = 0.049
φ and ω scans	θ_max_ = 25.0°, θ_min_ = 1.4°
Absorption correction: multi-scan (*SADABS*; Sheldrick, 1996)	*h* = −23→34
*T*_min_ = 0.952, *T*_max_ = 0.993	*k* = −5→5
2321 measured reflections	*l* = −7→7

### Refinement

**Table:**

Refinement on *F*^2^	Primary atom site location: structure-invariant direct methods
Least-squares matrix: full	Secondary atom site location: difference Fourier map
*R*[*F*^2^ > 2σ(*F*^2^)] = 0.039	Hydrogen site location: inferred from neighbouring sites
*wR*(*F*^2^) = 0.094	H-atom parameters constrained
*S* = 0.96	*w* = 1/[σ^2^(*F*_o_^2^) + (0.0524*P*)^2^] where *P* = (*F*_o_^2^ + 2*F*_c_^2^)/3
872 reflections	(Δ/σ)_max_ < 0.001
123 parameters	Δρ_max_ = 0.13 e Å^−^^3^
1 restraint	Δρ_min_ = −0.19 e Å^−^^3^

### Special details

**Table:**

Geometry. All e.s.d.'s (except the e.s.d. in the dihedral angle between two l.s. planes) are estimated using the full covariance matrix. The cell e.s.d.'s are taken into account individually in the estimation of e.s.d.'s in distances, angles and torsion angles; correlations between e.s.d.'s in cell parameters are only used when they are defined by crystal symmetry. An approximate (isotropic) treatment of cell e.s.d.'s is used for estimating e.s.d.'s involving l.s. planes.
Refinement. Refinement of *F*^2^ against ALL reflections. The weighted *R*-factor *wR* and goodness of fit *S* are based on *F*^2^, conventional *R*-factors *R* are based on *F*, with *F* set to zero for negative *F*^2^. The threshold expression of *F*^2^ > σ(*F*^2^) is used only for calculating *R*-factors(gt) *etc*. and is not relevant to the choice of reflections for refinement. *R*-factors based on *F*^2^ are statistically about twice as large as those based on *F*, and *R*- factors based on ALL data will be even larger.

### Fractional atomic coordinates and isotropic or equivalent isotropic displacement parameters (Å^2^)

**Table:**

	*x*	*y*	*z*	*U*_iso_*/*U*_eq_	Occ. (<1)
N1	0.93031 (9)	0.2933 (7)	0.6567 (4)	0.0497 (7)	
N2	0.79744 (9)	1.2194 (6)	0.1169 (4)	0.0479 (7)	
O1	0.94454 (7)	0.1141 (6)	0.8289 (3)	0.0560 (7)	
O2	0.76377 (8)	1.3375 (6)	0.1705 (3)	0.0643 (7)	
O3	0.81103 (8)	1.2669 (6)	−0.0530 (3)	0.0656 (8)	
C1	0.98599 (10)	−0.0342 (8)	0.8012 (4)	0.0508 (9)	
H1A	1.0106	0.0956	0.7815	0.061*	
H1B	0.9799	−0.1551	0.6775	0.061*	
C2	1.0000	−0.2040 (12)	1.0000	0.0511 (12)	
H2A	0.9741	−0.3236	1.0203	0.061*	0.50
H2B	1.0259	−0.3236	0.9797	0.061*	0.50
C3	0.89337 (11)	0.4213 (8)	0.6853 (5)	0.0486 (9)	
H3	0.8809	0.3840	0.8082	0.058*	
C4	0.86975 (10)	0.6240 (8)	0.5337 (4)	0.0418 (8)	
C5	0.88617 (10)	0.6955 (7)	0.3454 (4)	0.0501 (9)	
H5	0.9130	0.6115	0.3120	0.060*	
C6	0.86237 (10)	0.8918 (7)	0.2086 (5)	0.0481 (9)	
H6	0.8733	0.9422	0.0841	0.058*	
C7	0.82254 (10)	1.0105 (7)	0.2594 (4)	0.0405 (7)	
C8	0.80565 (10)	0.9443 (8)	0.4438 (4)	0.0470 (9)	
H8	0.7787	1.0280	0.4756	0.056*	
C9	0.82958 (10)	0.7517 (8)	0.5798 (4)	0.0473 (8)	
H9	0.8186	0.7061	0.7053	0.057*	

### Atomic displacement parameters (Å^2^)

**Table:**

	*U*^11^	*U*^22^	*U*^33^	*U*^12^	*U*^13^	*U*^23^
N1	0.0561 (17)	0.0467 (17)	0.0439 (14)	−0.0031 (16)	0.0009 (12)	0.0075 (15)
N2	0.0530 (16)	0.0421 (18)	0.0486 (14)	−0.0031 (15)	0.0076 (13)	0.0048 (14)
O1	0.0543 (14)	0.0622 (16)	0.0514 (12)	0.0090 (13)	0.0083 (10)	0.0130 (13)
O2	0.0691 (15)	0.0610 (18)	0.0650 (14)	0.0177 (15)	0.0178 (12)	0.0114 (14)
O3	0.0766 (15)	0.0688 (19)	0.0547 (12)	0.0025 (15)	0.0208 (11)	0.0196 (15)
C1	0.0476 (17)	0.047 (2)	0.0561 (19)	−0.0001 (18)	0.0044 (15)	−0.0014 (17)
C2	0.049 (3)	0.044 (3)	0.057 (3)	0.000	−0.001 (2)	0.000
C3	0.0467 (17)	0.051 (2)	0.0488 (18)	−0.002 (2)	0.0108 (15)	0.0082 (18)
C4	0.0441 (17)	0.0393 (19)	0.0408 (15)	−0.0048 (16)	0.0034 (14)	0.0022 (16)
C5	0.0471 (18)	0.056 (3)	0.0495 (17)	0.0035 (19)	0.0136 (15)	0.0007 (18)
C6	0.0535 (19)	0.052 (2)	0.0405 (16)	−0.0020 (19)	0.0125 (14)	0.0062 (16)
C7	0.0457 (16)	0.0351 (18)	0.0395 (15)	−0.0039 (15)	0.0032 (13)	0.0016 (14)
C8	0.0469 (18)	0.048 (2)	0.0486 (18)	0.0014 (18)	0.0165 (15)	0.0034 (17)
C9	0.0480 (17)	0.051 (2)	0.0448 (16)	0.0022 (18)	0.0139 (13)	0.0090 (17)

### Geometric parameters (Å, °)

**Table:**

N1—C3	1.273 (4)	C3—C4	1.459 (4)
N1—O1	1.401 (3)	C3—H3	0.9300
N2—O2	1.223 (3)	C4—C9	1.388 (4)
N2—O3	1.229 (3)	C4—C5	1.399 (4)
N2—C7	1.464 (4)	C5—C6	1.387 (4)
O1—C1	1.431 (3)	C5—H5	0.9300
C1—C2	1.503 (5)	C6—C7	1.371 (4)
C1—H1A	0.9700	C6—H6	0.9300
C1—H1B	0.9700	C7—C8	1.378 (4)
C2—C1^i^	1.503 (5)	C8—C9	1.374 (5)
C2—H2A	0.9700	C8—H8	0.9300
C2—H2B	0.9700	C9—H9	0.9300
			
C3—N1—O1	109.5 (2)	C4—C3—H3	118.5
O2—N2—O3	122.8 (3)	C9—C4—C5	119.0 (3)
O2—N2—C7	119.0 (2)	C9—C4—C3	118.4 (3)
O3—N2—C7	118.2 (3)	C5—C4—C3	122.7 (3)
N1—O1—C1	110.9 (2)	C6—C5—C4	119.9 (3)
O1—C1—C2	106.5 (2)	C6—C5—H5	120.0
O1—C1—H1A	110.4	C4—C5—H5	120.0
C2—C1—H1A	110.4	C7—C6—C5	119.2 (3)
O1—C1—H1B	110.4	C7—C6—H6	120.4
C2—C1—H1B	110.4	C5—C6—H6	120.4
H1A—C1—H1B	108.6	C6—C7—C8	122.1 (3)
C1^i^—C2—C1	114.5 (5)	C6—C7—N2	119.5 (3)
C1^i^—C2—H2A	108.6	C8—C7—N2	118.4 (3)
C1—C2—H2A	108.6	C9—C8—C7	118.5 (3)
C1^i^—C2—H2B	108.6	C9—C8—H8	120.7
C1—C2—H2B	108.6	C7—C8—H8	120.7
H2A—C2—H2B	107.6	C8—C9—C4	121.3 (3)
N1—C3—C4	122.9 (3)	C8—C9—H9	119.3
N1—C3—H3	118.5	C4—C9—H9	119.3
			
C3—N1—O1—C1	179.8 (3)	C5—C6—C7—N2	179.5 (3)
N1—O1—C1—C2	176.2 (3)	O2—N2—C7—C6	−175.2 (3)
O1—C1—C2—C1^i^	−64.9 (2)	O3—N2—C7—C6	5.4 (4)
O1—N1—C3—C4	179.9 (3)	O2—N2—C7—C8	3.4 (4)
N1—C3—C4—C9	179.9 (3)	O3—N2—C7—C8	−176.0 (3)
N1—C3—C4—C5	−0.6 (5)	C6—C7—C8—C9	−0.3 (5)
C9—C4—C5—C6	0.1 (5)	N2—C7—C8—C9	−178.9 (3)
C3—C4—C5—C6	−179.4 (3)	C7—C8—C9—C4	−0.4 (5)
C4—C5—C6—C7	−0.9 (5)	C5—C4—C9—C8	0.5 (5)
C5—C6—C7—C8	1.0 (5)	C3—C4—C9—C8	−179.9 (3)

Symmetry codes: (i) −*x*+2, *y*, −*z*+2.

### Hydrogen-bond geometry (Å, °)

**Table:**

*D*—H···*A*	*D*—H	H···*A*	*D*···*A*	*D*—H···*A*
C3—H3···O3^ii^	0.93	2.40	3.206 (4)	145
C9—H9···O3^ii^	0.93	2.63	3.395 (4)	139
C9—H9···O2^iii^	0.93	2.71	3.374 (4)	129

Symmetry codes: (ii) *x*, *y*−1, *z*+1; (iii) −*x*+3/2, *y*−1/2, −*z*+1.
